# Post-transplant cyclophosphamide plus anti-thymocyte globulin decreased serum IL-6 levels when compared with post-transplant cyclophosphamide alone after haploidentical hematopoietic stem cell transplantation

**DOI:** 10.1007/s44313-024-00049-z

**Published:** 2025-01-15

**Authors:** Jeong Suk Koh, Myung-Won Lee, Thi Thuy Duong Pham, Bu Yeon Heo, Suyoung Choi, Sang-Woo Lee, Wonhyoung Seo, Sora Kang, Seul Bi Lee, Chul Hee Kim, Hyewon Ryu, Hyuk Soo Eun, Hyo-Jin Lee, Hwan-Jung Yun, Deog-Yeon Jo, Ik-Chan Song

**Affiliations:** 1 Division of Hematology and Oncology, Department of Internal Medicine, Chungnam National University Hospital, 282 Munwha-Ro, Jung-Gu, Daejeon, 35015 South Korea; 2 Department of Medical Science, Daejeon, South Korea; 3 Brain Korea 21 FOUR Project for Medical Science, College of Medicine, Chungnam National University, Daejeon, South Korea

**Keywords:** IL-6, Post-transplantation cyclophosphamide, Anti-thymocyte globulin, GVHD, Hematopoietic stem cell transplantation

## Abstract

**Background:**

Post-transplantation cyclophosphamide (PTCy) and anti-thymocyte globulin (ATG) are common prophylactic strategies for graft-versus-host disease (GVHD) after haploidentical hematopoietic stem cell transplantation (haplo-HSCT). Interleukin (IL)-6 is a surrogate marker for cytokine release syndrome (CRS) and acute GVHD.

**Method:**

The clinical outcomes and complications of haplo-HSCT with PTCy plus ATG versus PTCy monotherapy were compared according to serum IL-6 levels at Chungnam National University Hospital (Daejeon, South Korea) from January 2019 to February 2023.

**Results:**

Forty patients who underwent haplo-HSCT were analyzed. A significant difference in IL-6 levels was observed between the PTCy plus ATG and PTCy alone groups (7.47 ± 10.55 vs. 117.65 ± 127.67; *p* = 0.003). More patients in the PTCy plus ATG group had a CRS grade of 0 than in the PTCy alone group (*p* < 0.001). Serum IL-6 levels were associated with grades II–IV acute GVHD (r = 0.547, *p* < 0.001). The cumulative incidence (CI) of grades II–IV acute GVHD was significantly higher in the PTCy alone group (67.9% vs. 4.8%; *p* < 0.001). No significant difference in the CI for chronic GVHD was detected between the PTCy plus ATG and PTCy alone groups (72.1% vs. 82.0%; *p* = 0.730). The CI of 1-year non-relapse mortality was significantly higher in the PTCy alone group than in the PTCy plus ATG group (42.2% vs. 15.9%; *p* = 0.022). The 1-year overall survival (OS) was significantly better in the PTCy plus ATG group (75.9% vs. 35.3%; *p* = 0.011). The 1-year GVHD-free, relapse-free survival rate was 29.4% in the PTCy alone group and 54.0% in the PTCy plus ATG group (*p* = 0.038).

**Conclusion:**

Serum IL-6 levels were higher in the PTCy alone group than in the PTCy plus ATG group. The addition of ATG before stem cell infusion affected IL-6 levels and reduced the incidences of CRS and grade II–IV acute GVHD in haplo-HSCT patients. This study suggests that PTCy plus ATG as GVHD prophylaxis in haplo-HSCT is beneficial in terms of clinical outcomes and complications of HSCT.

## Introduction

Allogeneic hematopoietic stem cell transplantation (HSCT) is an essential treatment for blood cancer [1]. Recently, haplo-identical HSCT (haplo-HSCT) has become widely used, with consistent improvements in treatment outcomes [2, 3]. Consequently, donor availability is no longer a major obstacle in allogeneic HSCT [4]. Nevertheless, graft-versus-host disease (GVHD) remains a major concern in the implementation of haplo-HSCT [5, 6]. Despite recent advances in GVHD treatment, 10–20% patients experience severe acute GVHD after allogeneic HSCT, and one-third of patients develop extensive chronic GVHD [7, 8]. Such severe grades of acute and chronic GVHD constitute a significant percentage of non-relapse mortality (NRM) and cause long-term sequelae that profoundly affect a patient’s quality of life. Therefore, prevention of severe GVHD is crucial for improving the clinical outcomes of haplo-HSCT [9]. Currently, the most commonly used regimens for GVHD prevention in T-cell-replete haplo-HSCT are anti-thymocyte globulin (ATG) and post-transplantation cyclophosphamide (PTCy) [7, 10]. ATG, which contains antibodies that primarily destroy T-cells, prevents GVHD in vivo via T-cell depletion [11]. PTCy effectively eliminates donor-derived alloreactive T-cells after allogeneic HSCT and preserves hematopoietic stem cells and regulatory T-cells, thereby demonstrating its preventive effects against GVHD [12, 13].

No prospective studies have compared the efficacies of PTCy and ATG in haplo-HSCT. A retrospective analysis using data from the European Society for Blood and Bone Marrow Transplantation (EBMT) registry showed that a PTCy-based regimen led to better outcomes than an ATG-based regimen in patients with acute myeloid leukemia [7]. The PTCy-based regimen resulted in longer durations of leukemia-, GVHD-, and relapse-free survival, along with lower incidences of GVHD and NRM. Furthermore, in a recent meta-analysis, a PTCy-based regimen demonstrated superior overall survival (OS) and GVHD prevention than an ATG-based regimen, even in unrelated-donor allogeneic HSCT [14]. Therefore, PTCy is considered a new standard for GVHD prophylaxis in haplo-HSCT and unrelated-donor HSCT [15, 16].

Despite the use of PTCy-based regimens, some patients experience severe GVHD or cytokine release syndrome (CRS), occasionally leading to life-threatening complications [17, 18]. Previously, we reported that 10–20% patients receiving a PTCy-based regimen experienced severe CRS and acute GVHD, resulting in serious neurological complications such as autoimmune limbic encephalitis [19]. Therefore, to reduce the incidences of CRS and severe acute GVHD, clinical trials combining PTCy and ATG have recently been conducted [14, 20, 21]. Additionally, interleukin-6 (IL-6) has been investigated as a biomarker for predicting acute GVHD or CRS in haplo-HSCT by using PTCy [22, 23]. To date, no study has compared IL-6 levels between groups treated with PTCy alone vs. PTCy plus ATG. The aim of this study was to analyze the correlations between IL-6 levels and clinical outcomes in both groups.

## Materials and methods

### Patients and treatments

We retrospectively analyzed consecutive adults (age, > 18 years) with hematological malignancies who underwent human leukocyte antigen (HLA) haploidentical donor allogeneic HSCT at Chungnam National University Hospital (CNUH; Daejeon, South Korea) between January 2019 and February 2023. PTCy (50 mg/kg) was administered on days + 3 and + 4. Rabbit ATG (thymoglobulin, 1.5 mg/kg; Sanofi-Aventis, Paris, France) was administered from day 3 to 1. The regimen included tacrolimus beginning on day + 5, with a target level of 5–15 ng/mL. Mycophenolate mofetil, up to 3 g/day in divided doses, was administered from day 5 to day 35. Two conditioning regimens were administered. In the myeloablative conditioning (MAC) regimen, 3.2 mg/kg busulfan was administered for 4 days and 30 mg/m^2^ fludarabine was administered for 5 days. In the reduced-intensity conditioning (RIC) regimen, 3.2 mg/kg busulfan was administered for 2 days and 30 mg/m^2^ fludarabine was administered for 5 days. RIC was administered to patients of > 55 years of age or those with comorbidities. The busulfan dose was not pharmacokinetically adjusted. All patients received granulocyte colony-stimulating factor-mobilized peripheral blood stem cells (PBSCs; target CD34 + cell count, 5 × 10^6^/kg). Filgrastim (300 μg/m^2^) was administered from day + 5 until neutrophil recovery. No therapy such as donor lymphocyte infusion, hypomethylating agents, or tyrosine kinase inhibitors, was administered to prevent relapse after allogeneic HSCT.

### Study end points and definitions

The primary study outcome was the difference in IL-6 levels and their correlations with acute GVHD between PTCy alone and PTCy plus ATG treatment groups. The secondary outcomes were OS, incidence and severity of acute and chronic GVHD, leukemia-free survival, relapse rate, NRM, CRS, veno-occlusive disease (VOD), cytomegalovirus (CMV), and Epstein–Barr virus (EBV) reactivation in each group. Acute GVHD was graded using the Mount Sinai Acute GVHD International Consortium (MAGIC) criteria, and chronic GVHD was graded according to the National Institutes of Health (NIH) consensus [24, 25]. GVHD-free, relapse-free survival (GFRS) was defined as the occurrence of any of the following events from the time of transplantation: grade III or IV acute GVHD, chronic GVHD warranting systemic immunosuppression, disease relapse or progression, or death from any cause. NRM was defined as death from any cause except relapse. CRS was graded using the American Society for Transplantation and Cellular Therapy scale [26]. All CRS manifestations occurred within the first 6 days after transplantation. CMV and EBV reactivation was defined as the detection of viral DNA in whole blood by using PCR on at least one occasion.

### Sample collection and analysis

Peripheral blood samples were collected from the patients on day + 3 after haplo-HSCT. Serum IL-6 levels were measured using the chemiluminescent immunoassay (CLIA) with an Elecsys IL-6 kit (Roche Diagnostics, Mannheim, Germany) in a clinical laboratory at CNUH. In accordance with the manufacturer’s instructions, serum IL-6 reference values were set to 0–7.0 pg/mL.

### Statistical analysis

Categorical variables were compared using the chi-square test, and logistic regression was performed to examine correlations. Overall and leukemia-free survival durations were assessed using the Kaplan–Meier method. Survival rates were compared using the log-rank test. Cumulative incidence (CI) functions were used to estimate the rates of acute and chronic GVHD, relapse rate, and NRM. A *p*-value < 0.05 was considered statistically significant. Cox proportional hazards regression was used to evaluate OS. Clinically relevant factors with a *p*-value < 0.05 in univariate analyses were used in the multivariate analysis. All statistical analyses were performed using SPSS ver. 24.0 software (IBM, Armonk, NY, USA).

## Results

### Patient and transplantation characteristics

The study included 40 patients undergoing haplo-HSCT divided into two groups on the basis of the GVHD prophylaxis received: PTCy plus ATG (*n* = 23) or PTCy alone (*n* = 17). The baseline characteristics are summarized in Table 1. The median age at diagnosis was 60 and 56 years, respectively. No significant differences were observed between the groups in terms of sex, disease type, conditioning intensity, poor-risk disease, or HCT-CI. Although the difference was not significant, the MAC regimen was administered to a greater percentage of patients in the PTCy-alone group (47.1% vs. 30.4%; *p* = 0.336). No difference in EBV reactivation was observed between the two groups. The CMV reactivation rate was significantly higher in the PTCy-alone group (82.4% vs. 26.1%, *p* < 0.001). In the PTCy plus ATG group, 9 of 23 (39.0%) patients received letermovir, when compared with none in the PTCy alone group (*p* = 0.005). The incidence of VOD was significantly higher in the PTCy alone group than in the PTCy plus ATG group (24.0% vs. 0.0%, *p* = 0.026). Grade III/IV acute GVHD occurred more frequently in the PTCy alone group than in the PTCy plus ATG group (26.7% vs. 0.0%, *p* = 0.029). The total number of patients without CRS was higher in the PTCy plus ATG group than in the PTCy alone group (87.0% vs. 5.9%, *p* < 0.001). The median follow-up durations in the PTCy plus ATG and PTCy alone groups were 9.3 (range 3.3–22.8) and 5.5 (range 0.3–40.5) months, respectively.

**Table 1:** Clinical characteristics between PTCy with ATG and PTCy groups underwent haplo-HSCT (*n* = 40)

	PTCy + ATG (*n* = 23)	PTCy (*n* = 17)	*p*-value
Median Age, year (range)	60, (18–71)	56, (28–72)	0.997
Gender, M: F	12: 11	10: 7	0.755
Type of diseases			0.476
AML	16 (69.6%)	12 (70.6%)	
ALL	3 (13.0%)	4 (23.5%)	
MDS & PMF	4 (17.4%)	1 (5.9%)	
Conditioning intensity			0.336
MAC	7 (30.4%)	8 (47.1%)	
RIC	16 (69.6%)	9 (52.9%)	
Disease status at transplant			0.053
1st CR	19 (82.6%)	12 (70.6%)	
2nd CR	0 (0.0%)	3 (17.6%)	
3rd CR	0 (0.0%)	1 (5.9%)	
MDS & PMF	4 (17.4%)	1 (5.9%)	
Poor risk^a^	12 (60.0%)	10 (62.5%)	1.000
HCT-CI			0.389
0	15 (65.2%)	12 (70.6%)	
1–2	5 (21.7%)	5 (29.4%)	
3-	3 (13.0%)	0 (0.0%)	
EBV reactivation	2 (8.7%)	0 (0.0%)	0.499
CMV reactivation	6 (26.1%)	14 (82.4%)	< 0.001
CMV prophylaxis with letermovir	9 (39.0%)	0 (0.0%)	0.005
VOD incidence	0 (0.0%)	4 (24.0%)	0.026
Acute GVHD (evaluable)			0.029
None	15 (65.2%)	6 (40.0%)	
Grade I/II	8 (34.8%)	5 (33.3%)	
Grade III/IV	0 (0.0%)	4 (26.7%)	
Stem cell source			-
PB	23 (100%)	17 (100%)	
BM	0 (0.0%)	0 (0.0%)	
CRS grading			< 0.001
No CRS	20 (87.0%)	1 (5.9%)	
Grade 1	3 (13.0%)	10 (58.8%)	
Grade 2	0 (0.0%)	6 (35.3%)	
Cell count, median (range)			
TNC count (× 10^8^ cells/kg)	13.90 (6.09–23.28)	13.18 (6.41–21.71)	0.576
CD34 + cell (x10^6^cells/kg)	8.56 (4.50–17.77)	10.68 (2.88–25.85)	0.224
Median F/U duration, month (range)	5.5 (0.3–40.5)	9.3 (3.3–22.8)	0.255

*PBSCT *Peripheral blood stem cell transplantation, *CR *Complete remission, *HCT-CI *Hematopoietic stem cell transplantation comorbidity index, *CMV *Cytomegalovirus, *VOD *Veno occlusive disease, *PB *Peripheral blood, *BM *Bone marrow, *MAC *Myeloablating conditioning, *RIC *Reduced intensity conditioning, *TNC *Total nucleated cell

^a^Poor risk includes sAML, tAML, AML with poor risk group in NCCN guideline, poor cytogenetics in ALL

### IL-6 levels and acute GVHD and CRS

**Fig. 1:**
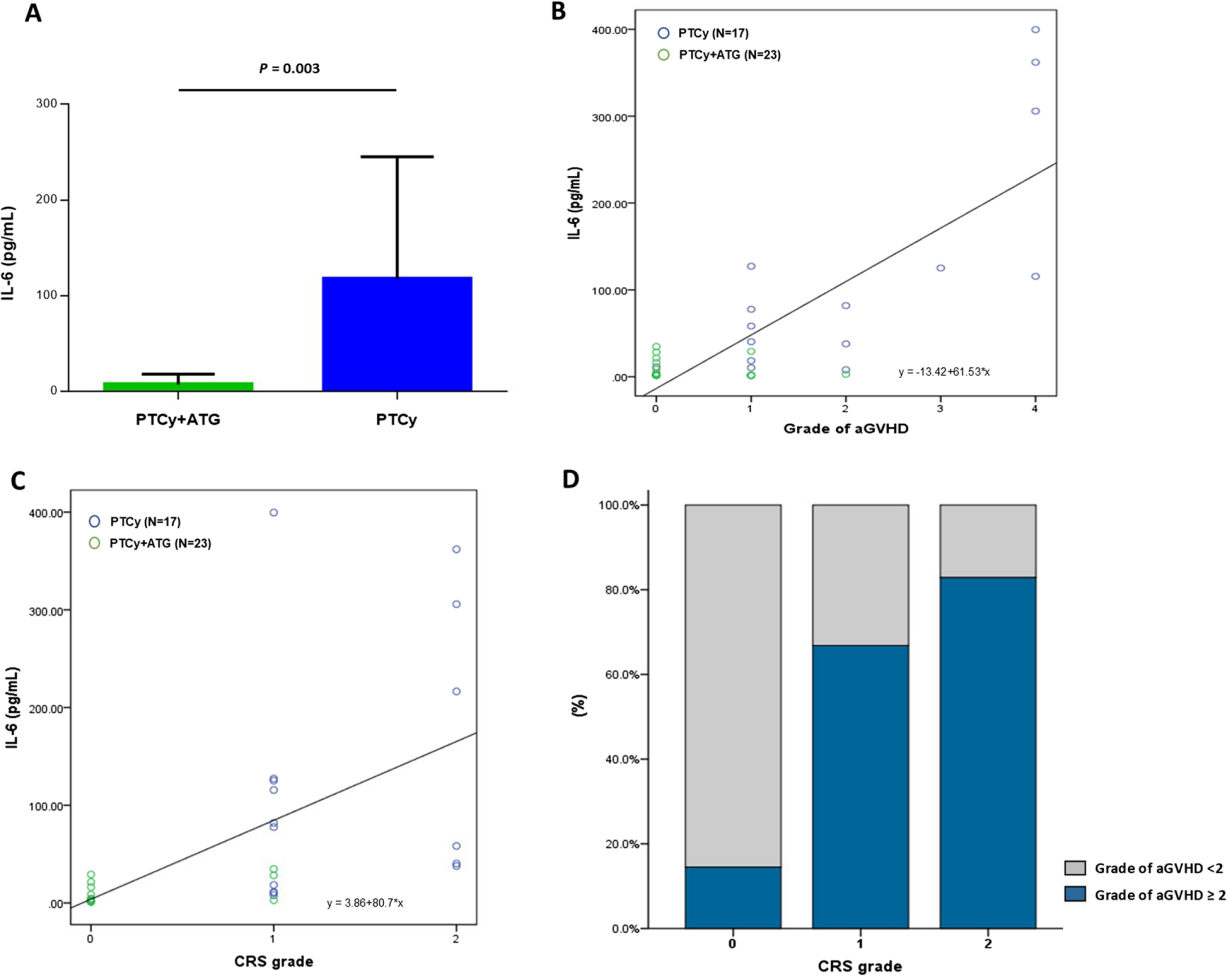
IL-6 Levels, acute GVHD, and CRS. **A** Plasma IL-6 levels between PTCy plus ATG and PTCy alone groups before PTCy infusion. **B** Association between severity of acute GVHD and plasma IL-6 levels. **C** Association between CRS grade and plasma IL-6 levels. **D** The percentage of patients who developed grade 2 or higher acute GVHD according to the CRS grade

A significant difference in IL-6 levels was observed between the PTCy plus ATG and PTCy alone groups (7.47 ± 10.55 vs. 117.65 ± 127.67, respectively; *p* = 0.003, Fig. 1A). IL-6 levels increased concurrently with the severity of acute GVHD (r = 0.547, *p* < 0.001; Fig. 1B). CRS severity was correlated with IL-6 levels (r = 0.801, *p* < 0.001, Fig. 1C) and positively correlated with the degree of acute GVHD (*p* = 0.004, Fig. 1D).

### GVHD and CRS between PTCy with ATG and PTCy alone

**Fig. 2:**
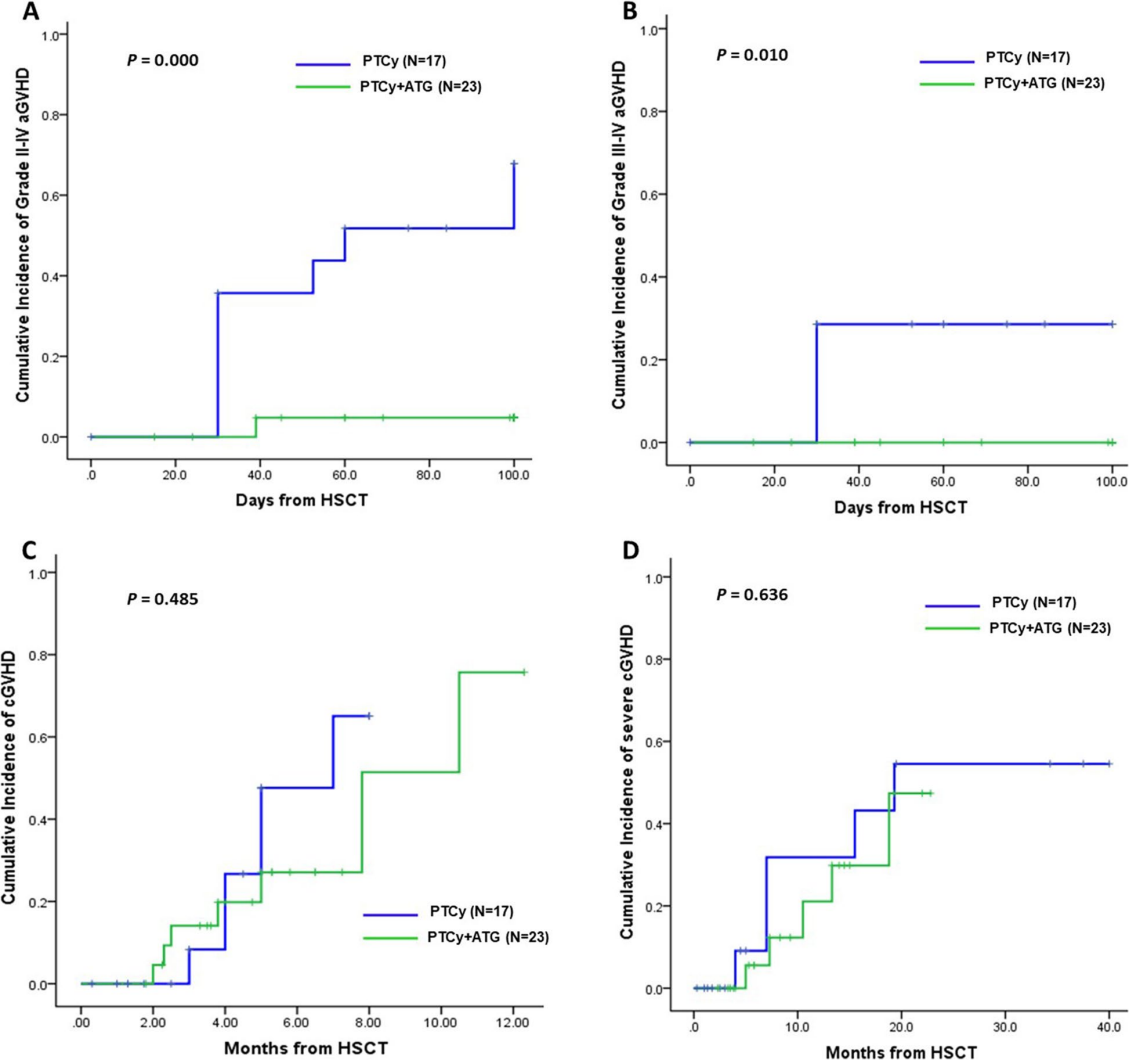
Cumulative incidences of GVHD between PTCy plus ATG and PTCy alone. **A** The CI of grade II–IV acute GVHD. **B** The CI of grade III-IV acute GVHD. **C** The CI of chronic GVHD. **D** The CI of severe chronic GVHD

The CI of grade II-IV acute GVHD on day 100 was significantly higher in the PTCy alone group than in the PTCy plus ATG group (67.9% vs. 4.8%; *p* < 0.001; Fig. 2A). PTCy alone recipients were more likely to develop grade III/IV acute GVHD than PTCy plus ATG recipients (26.7% *vs*. 0.0%, respectively; *p* = 0.010, Fig. 2B). No significant differences were detected between the PTCy plus ATG and PTCy alone groups in the 1-year CI of chronic GVHD (75.7% vs. 65.1%; *p* = 0.485; Fig. 2C) or 1-year CI of severe chronic GVHD (12.3% vs. 43.2%; *p* = 0.636; Fig. 2D).

Fever occurred within 6 days after stem cell infusion in 22 of 40 patients (55%). A regular pattern of fever was noted, which generally disappeared within 48 h after the initial cyclophosphamide administration and fully resolved in all patients by day + 7. Patients in the PTCy alone group were more likely to develop grade 2 CRS than those in the PTCy plus ATG group (35.3% vs. 0.0%; *p* < 0.001).

### Survival outcomes

**Fig. 3:**
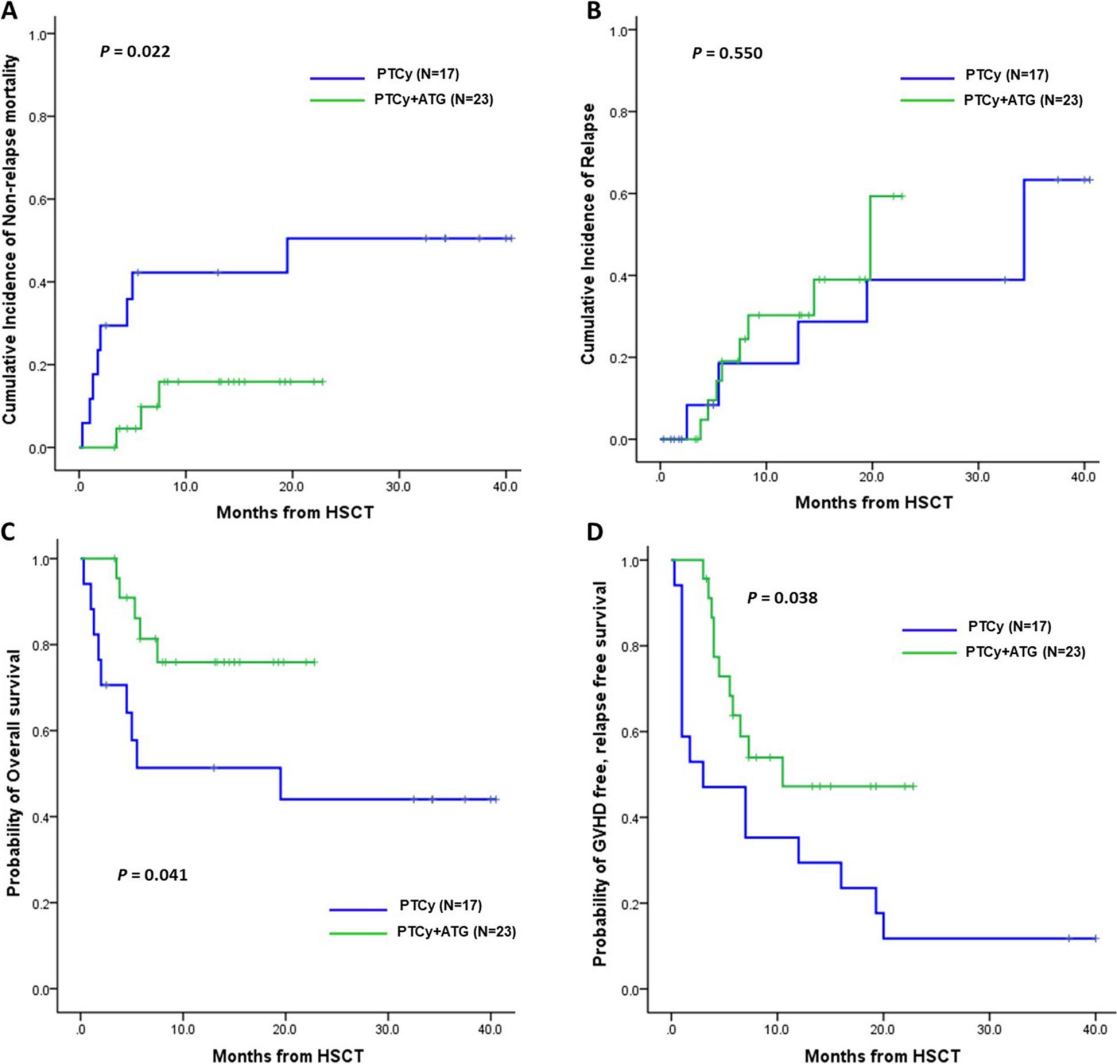
Clinical outcomes between PTCy plus ATG and PTCy alone. **A** The CI of non-relapse mortality (NRM). **B** The CI of relapse. **C** The probability of overall survival (OS). **D** The probability of GVHD-free, relapse-free survival (GFRS)

The CI of 1-year NRM was significantly higher in the PTCy alone group (42.2% vs. 15.9%; *p* = 0.022; Fig. 3A), with no significant difference in 1-year relapse mortality between the two groups (PTCy plus ATG, 30.3% vs. PTCy alone, 28.5%; *p* = 0.550; Fig. 3B). The causes of NRM included infection, GVHD, and intracranial hemorrhage. The 1-year OS was significantly higher in the PTCy plus ATG group than in the PTCy alone group (75.9% vs. 44.0%; *p* = 0.041; Fig. 3C). The 1-year GFRS rate was 29.4% in the PTCy alone group and 54.0% in the PTCy plus ATG group (*p* = 0.038; Fig. 3D).

Next, we performed univariate and multivariate analyses of OS and NRM (Tables 2 and 3, respectively). Univariate analysis showed that non-CR1 status at transplantation, grade II-IV acute GVHD, and serum IL-6 levels were associated with poor OS. Multivariate analysis confirmed that IL-6 levels were an independent adverse risk factor for OS in patients who underwent haplo-HSCT (hazard ratio [HR] = 47.462, *p* = 0.002).

**Table 2:** Univariate and Multivariate analysis for risk factors of OS

Variables	Univariate analysis		Multivariate analysis	
	*p* value	HR (95% CI)	*p* value	HR (95% CI)
Age ≥ 57 vs < 57	0.254	1.912 (0.628–5.821)		
GVHD prophylaxis				
PTCy + ATG vs. PTCy	0.051	0.333(0.110–1.006)		
Conditioning intensity				
Myeloablative vs Reduced intensity	0.124	2.765 (0.757–10.097)		
HCT-CI score ≥ 3 vs. 0–2	0.957	0.945 (0.120–7.438)		
Non-CR1 status at transplant	0.011	5.830 (1.504–22.592)	0.233	3.178 (0.475–21.285)
Cytogenetic Risk status at diagnosis				
Poor vs. favorable or intermediate	0.999	1.001 (0.491–2.040)		
Acute GVHD II-IV vs. 0-I	0.001	7.986 (2.473–25.788)	0.283	2.632 (0.450–15.379)
Severe chronic GVHD vs. Non-severe	0.239	0.406 (0.090–1.823)		
IL-6 levels	< 0.001	1.009 (1.005–1.014)	0.011	1.009 (1.002–1.016)
CRS severity: grade 2 or greater vs. 0 or 1	0.238	2.173 (0.599–7.891)		

*Tx* Treatment, *OS* Overall survival, *GVHD* Graft versus host disease, *HCT-CI* Hematopoietic stem cell transplantation comorbidity index, *CR* Complete remission, *CRS* Cytokine release syndrome

**Table 3:** Univariate and Multivariate analysis for risk factors of NRM

Variables	Univariate analysis		Multivariate analysis	
	*p* value	HR (95% CI)	*p* value	HR (95% CI)
Age ≥ 57 vs. < 57	0.318	1.897 (0.540–6.664)		
GVHD prophylaxis				
PTCy + ATG vs. PTCy	0.034	0.235 (0.061–0.898)	0.453	2.470 (0.233–26.216)
Conditioning intensity				
Myeloablative vs Reduced intensity	0.118	3.470 (0.730–16.486)		
HCT-CI score ≥ 3 vs. 0–2	0.788	1.329 (0.168–10.523)		
Non-CR1 status at transplant	0.070	4.517 (0.885–23.047)		
Cytogenetic Risk status at diagnosis				
Poor vs. favorable or intermediate	0.586	1.293 (0.512–3.266)		
Acute GVHD II-IV vs. 0-I	0.006	6.717 (1.748–25.805)	0.418	2.756 (0.237–32.113)
Severe chronic GVHD vs. Non-severe	0.187	0.250 (0.032–1.963)		
IL-6 levels	< 0.001	1.010 (1.005–1.014)	0.005	1.012 (1.004–1.020)
CRS severity: grade 2 or greater vs. 0 or 1	0.139	2.747 (0.720–10.489)		

*NRM* Non-relapse mortality, *Tx* Treatment, *OS* Overall survival, *GVHD* Graft versus host disease, *HCT-CI* Hematopoietic stem cell transplantation comorbidity index, *CR* Complete remission, *CRS* Cytokine release syndrome

The PTCy alone regimen of GVHD prophylaxis, grade II-IV acute GVHD, and IL-6 levels were associated with NRM in the univariate analyses (Table 3). Multivariate analysis revealed that IL-6 level was the only risk factor associated with NRM (HR = 1.012, *p* = 0.005).

## Discussion

Recently, several retrospective studies have evaluated the use of ATG plus PTCy to prevent GVHD [20, 21, 27]. However, no reports on the utility of IL-6 as an indicator of acute GVHD have been published in the context of haplo-HSCT using PTCy or PTCy plus ATG. In this study, we demonstrated that the addition of ATG to PTCy reduced post-transplant IL-6 levels, contributing to a lower severity of CRS and acute GVHD after haplo-HSCT, thereby improving survival outcomes.

IL-6 is a pro-inflammatory cytokine that serves as biomarker for CRS and acute GVHD after haplo-HSCT [18, 22]. The main source of IL-6 is monocyte lineage cells; monocytes are presumably stimulated to secrete IL-6 upon alloreactive T-cell activation after haplo-HSCT [28]. IL-6 inhibits transforming growth factor (TGF)-β-induced T-cell differentiation into regulatory T (Treg) cells and downregulates Foxp3 expression by Treg cells [29, 30]. Elevated IL-6 levels are suspected to reduce Treg activity, ultimately resulting in increased severity of acute GVHD. Previously, we demonstrated that the Treg population was reduced upon increase of post-transplant IL-6 levels in patients who underwent haplo-HSCT using PTCy. Moreover, a substantial decrease in the active subpopulation of Tregs was observed, which contributes to the onset of severe CRS and encephalopathy [19].

Greco et al. measured IL-6 levels on day 7 post-transplantation, whereas we measured it on day 3. We utilized this approach because our previous study serially analyzed IL-6 and other inflammatory cytokines after haplo-HSCT using PTCy and showed that the highest IL-6 values occurred on post-transplantation day 3 [19]. Furthermore, the peak clinical manifestation of CRS, primarily fever, occurred immediately before PTCy administration. Therefore, we measured IL-6 levels on post-transplantation day three and analyzed the clinical outcomes associated with IL-6 levels. Furthermore, in our study, IL-6 was the only predictor of survival and treatment-related mortality, suggesting that IL-6 measurements after haplo-HSCT with PTCy may be useful for predicting both acute GVHD and post-transplant prognosis.

In previous studies comparing PTCy plus ATG with PTCy alone, the addition of ATG to PTCy appeared to reduce the incidences of acute and chronic GVHD without increasing the rate of relapse [31–33]. Battipaglia et al. reported that PTCy plus ATG was associated with a lower risk of chronic GVHD than PTCy alone, without higher rates of transplantation toxicity, mortality, or relapse [31]. El-Cheikh et al. also reported that the addition of ATG to PTCy reduced the incidence of acute GVHD and increased OS in HSCT [32]. In our study, PTCy plus ATG significantly reduced the incidences of VOD and NRM, leading to improved OS. PTCy plus ATG did not affect relapse incidence. To investigate the mechanisms underlying these effects, Makanga et al. analyzed T- and NK cells in the peripheral blood of patients undergoing haplo-HSCT using PTCy and ATG; they suggested that slower T-cell reconstitution is involved in the reduced incidence of GVHD, whereas faster-recovering subtypes of NK cells help prevent relapse [33].

However, the optimal ATG dose for PTCy has not yet been determined. Several studies have investigated lower doses of ATG and PTCy [20, 21]. Xu et al. compared low-dose ATG (5 mg/kg) plus low-dose PTCy (one dose of 50 mg/kg) with the standard dose of ATG (10 mg/kg) and reported that low-dose ATG plus PTCy reduced GVHD risk and NRM [20]. A retrospective analysis of a large sample with long-term follow-up suggested that low-dose ATG/PTCy effectively prevented severe acute GVHD [34]. Wang et al. reported that low-dose PTCy (14.5 mg/kg on days 3 and 4) plus ATG reduced acute and chronic GVHD relative to the standard-dose ATG regimen [21]. Additionally, Kim et al. adjusted the ATG dose according to the absolute lymphocyte count on day 3 before haplo-HSCT and administered 80 mg of PTCy. They reported that the dual T-cell-depleting regimen improved survival when compared with ATG alone [35]. Intriguingly, they reported that the rate of life-threatening infections in the post-engraftment period was lower in the ATG/PTCy combination group than in the ATG alone group. Therefore, further research is required to determine the optimal dosages of ATG and PTCy when used in combination.

This study has some limitations. First, it was a retrospective, single-center study with a small number of patients. Thus, future multicenter studies with more patients are required to generalize our findings. However, this study analyzed consecutive patients treated using the same protocol at the same institution, which may have reduced selection bias. Second, the rate of CMV reactivation was higher in the PTCy alone group, contrary to the generally accepted notion that increased immunosuppressive therapy strength is associated with a greater likelihood of CMV reactivation. This may have occurred because national health insurance coverage for letermovir became available in South Korea in March 2021. However, there were no deaths due to CMV disease, indicating that this difference was unlikely to influence the OS.

In conclusion, the addition of ATG to PTCy decreased IL-6 levels; reduced the incidence of CRS, acute GVHD, and NRM; and improved OS. IL-6 levels measured 3 days after haplo-HSCT with PTCy can be used to predict OS, NRM, and GVHD.

## Data Availability

No datasets were generated or analysed during the current study.
